# Multidimensional Factors Affecting Successful Aging among Empty-Nesters in China Based on Social-Ecological System Theory

**DOI:** 10.3390/ijerph191911885

**Published:** 2022-09-20

**Authors:** Hui Chang, Jia Zhou, Zhiwen Wang

**Affiliations:** 1 School of Nursing, Guizhou Medical University, Guiyang 550025, China; 2 School of Nursing, Peking University, Beijing 100191, China

**Keywords:** associated factors, empty-nesters, social-ecological system, successful aging

## Abstract

Background: This study aims to identify the status of successful aging and the factors influencing empty-nest elderly in China based on the social-ecological system theory. Methods: The data came from the follow-up survey (2018) of the China Health and Retirement Longitudinal Study and 3074 empty-nesters aged 60 and over are included. Chi-squared tests and logistic regressions were used to identify factors associated with successful aging. Results: The successful aging rate of empty-nesters in China was 5.9%. The results of the multifactor analysis showed that younger age, higher education level, good self-rated health, good hearing, high life satisfaction, availability of financial resources at the microsystem level, higher frequency of contact with children at the mesosystem level, and medical insurance at the macrosystem level were the contributing influencing factors for successful aging of empty-nesters in China. Conclusion: This study is an important attempt to explore the successful aging of empty-nesters in China. Because this study is based on social-ecological system theory, it confirms the important role of individual characteristics of older adults and their surrounding environment in achieving successful aging. Therefore, we should pay attention not only to the individual characteristics of the elderly, but also to the role of the surrounding environment on the health of the elderly, so that we can develop intervention measures to promote their successful aging.

## 1. Introduction

The global population is aging, with 733 million people aged 65 or older in the world as of 2019 [1]. Moreover, China has the world’s largest elderly population [2]. The latest census data released in 2021 show that China’s population aged 60 and above is 264 million. Compared with 2010, the population aged 60 years in China has increased by 5.44 percentage points [3]. The evolving perspectives on aging have shifted from “how to live longer” to “how to age well”, and the definition of successful aging has been perceived as a useful tool for describing the health status of the elderly population [4]. Since the concept of successful aging was introduced in 1961 by Havighurt, it has been explored by many scholars [5,6,7,8], but there is still no unified definition. Currently, the Rowe and Kahn model [9] is widely used, which defines successful aging as the absence of disease and disability, good physical and cognitive functioning, and active social participation.

As the speed of change in the traditional family structure increases, as does the population flow, empty-nesters are becoming a large group of elderly people. Empty-nesters were defined as people aged 60 years and above who had no children or had children that were not around for long periods of time (more than one month), including elderly people living alone and with their spouses [10]. Data from the seventh census show that in 2021, the population of China’s average family household fell below three people for the first time. The size of family households continues to shrink, making the problem of empty nesting increasingly serious [3]. It is expected that the proportion of empty-nesters in China will reach 90% by 2030 [11], and more than 200 million people are expected to become empty-nesters [12]. Compared to non-empty-nesters, empty-nesters are more likely to face physical [13,14], psychological [12,15], and social problems [16,17] due to their health status, interpersonal communication, lifestyle, and social support. A meta-analysis showed that empty-nesters were more likely to suffer from depression than non-empty-nesters (44.2% vs. 26.3%) [15]. Therefore, under the context of aging, how to avoid the poor health of elderly empty-nesters and realize high-quality aging is an urgent issue that needs attention and exploration.

Successful aging is an important indicator of the health and aging status of older adults [18]. Although theories and practices related to successful aging are being explored in response to increasing global population aging, the level of successful aging is low and varies around the world due to differences in the definition and the influence of individual differences in elderly populations and environmental factors [18]. In a study conducted by Jang [8] in Korea, it was shown that the successful aging of empty-nesters is even less optimistic, where the successful aging rate of those living with a spouse was 16.4%, whereas the successful aging rate of those living alone was only 7.8%. Several studies have shown that sociodemographic characteristics [19,20,21] (e.g., gender, age, marital status, education, and employment status, etc.), health behavior factors [20,22] (e.g., alcohol drinking, exercise, etc.), income [21], relevant physical health conditions [18,20], and family and social factors [8] are important influence factors of successful aging. Huang [23] found through qualitative research that the successful aging of empty-nesters is influenced by subjective factors such as “self-esteem and self-worth” and “emotion” and objective factors such as “physiological health”, “economic situation”, and “medical security”, among others. In summary, the realization of successful aging may be related to individual, family, and social factors.

The social-ecological system theory was first proposed by American psychologist Bronfenbrenner [24], who placed people in their environment and emphasized the interaction between people and the environment. The American scholar Charles H Zastrow divided the social-ecological system into microsystems, mesosystems, and macrosystems [25]. Microsystems mainly refer to individual systems in the social environment, such as some physical and psychological characteristics of individuals. Mesosystems refer to small-scale groups that have an impact on individuals in the social environment, such as family, neighbors, and co-workers etc. Finally, a macrosystem includes community, country, and culture. The realization of successful aging of the elderly is not only related to individual factors of the elderly, but also closely related to the family and social environment in which they live. This also reflects the social-ecological theory [24] that individual behavior is affected by the social environment and its interaction with it, as well as the interaction and interaction among several systems (microsystem, mesosystem, and macrosystem) that are interconnected between individual development and surrounding environment [26].

Although there have been studies on successful aging and empty-nesters in China, most of them focus on the analysis of the current situation and influencing factors of successful aging in the community or elderly institutions. However, empty-nest elderly people are different from non-empty-nest elderly people in social areas and elderly people in institutions, most studies on this special group of empty-nesters only focus on the current situation and psychological condition of empty-nesters. Huang [23] studied the successful aging of empty-nesters through a qualitative study in economically developed Shanghai, so the results may not be representative, therefore there is no quantitative study on the successful aging of empty-nesters with nationally representative samples. Although some foreign countries have conducted studies on the successful aging of the elderly by considering various factors, their findings are available for reference but not suitable for replication in our country due to the differences in cultural background and social security system between China and foreign countries. The purpose of this study is to use a nationally representative database to focus on empty-nesters to explore the current situation of successful aging and the factors influencing the successful aging of empty-nesters in China at three levels: microsystem, mesosystem, and macrosystem, based on Charles H Zastrow ’s social-ecological system.

## 2. Materials and Methods

### 2.1. Study Design

A cross-sectional and quantitative design was employed. We followed the Reporting of Studies Conducted Using Observational Routinely Collected Health Data (RECORD) reporting guidelines, an extension of Strengthening the Reporting of Observational Studies in Epidemiology (STROBE) (Supplementary Table S1).

### 2.2. Data Source

The data of this study were from the follow-up survey of the China Health and Retirement Longitudinal Study (CHARLS), a large social survey project in China. The CHALRS project team started the national baseline survey in 2011 and selected 150 counties and 450 communities (villages) in 28 provinces for field visits. The database is available through the official website (http://charls.pku.edu.cn/, accessed on 23 April 2022). The CHARLS study gained approval for interviewing respondents and collecting data by the Biomedical Ethics Review Committee of Peking University (IRB00001052-11015).

The data used in this study were collected from wave 4 of the CHARLS in 2018, a survey of 19,816 people. According to the needs of this study, data related to the successful aging of empty-nest elderly people aged 60 and over were selected for analysis, and missing values were eliminated.

### 2.3. Variables and Instruments

#### 2.3.1. The Dependent Variable

In this study, successful aging was evaluated as a dependent variable. This paper defines successful aging according to the most cited Rowe & Kahn theoretical model [9], which mainly includes the following five points: (1) No main diseases; (2) No disability; (3) High cognitive function; (4) No depression; (5) Active engagement with life. If these conditions are fully met by the elderly, they have achieved successful aging. The indicators of successful aging are defined below:

(1) No main diseases: Participants were classified as having “No main disease” if they did not report they had been diagnosed with any of the following major diseases (cancer, chronic lung disease, diabetes, heart disease, and stroke) by doctors.
(2) No disability: Respondents were classified as not having any disability according to the ADL scale if they reported no difficulty in performing the following activities of daily living: dressing, bathing, eating, getting in and out of bed, going to the toilet, and controlling urine and feces.
(3) High cognitive function: The cognitive function of the respondents was evaluated by daily memory, word recall, arithmetic ability, and drawing ability, with a total score range of 0–21. Respondents who scored below the mean score were considered to have impaired cognitive functioning [22].
(4) No depression: Depressive symptoms were assessed using the CES-D 10(10-item Center for Epidemiological Studies Depression Scale) [27], which has been used for measuring older adults’ depressive symptoms and has been shown to be effective in China [28]. The total score of the scale ranges from 0 to 30, with a lower score indicating a lower level of depressive symptoms. A cut-off score of ≥10 was used to identify the respondents who had depressive symptoms significantly [29].
(5) Active engagement with life: Respondents were defined as being actively engaged if they reported participation in “voluntary or charity work,” “having provided help to family, friends or neighbors,” or having “gone to a sport, social or other kind of club” in the month preceding the interview.

#### 2.3.2. The Independent Variable

The factors within the scope of the theory of social ecosystem were evaluated as follows:

(1) Microsystem-related factors: The microsystem level included the individual characteristics and behaviors of the elderly. Personal characteristics include gender, age, education level, self-assessment of health, the satisfaction of life, financial income, vision and hearing, and personal behavior including whether the elderly smoke, drink alcohol and exercise, and sleep time (Table 1).
(2) Mesosystem-related factors: The variables for the mesosystem included the marital status, number of children, number of siblings, financial support received from children, financial support to children, contact with the child, satisfaction with children, and satisfaction with the spouse, and living arrangement (Table 1).
(3) Macrosystem-related factors: The macrosystem included ethnicity, religion, community type, medical insurance, pension, community elderly care service, and medical aid (Table 1).

### 2.4. Data Analysis

SPSS 25 was used for data analysis. Descriptive statistics were used to show the respondents’ sociodemographic characteristics. A χ2-test was applied as the comparison of categorical variables. Multiple logistic regression analysis was applied to estimate the odds ratio (OR) for successful aging based on predictor variables. The level of significance was set at 95% (*p* < 0.05).

## 3. Results

### 3.1. Demographic Characteristics and the Effects of the Microsystem, Mesosystem, and Macrosystem Variables on Successful Aging

**Figure 1:**
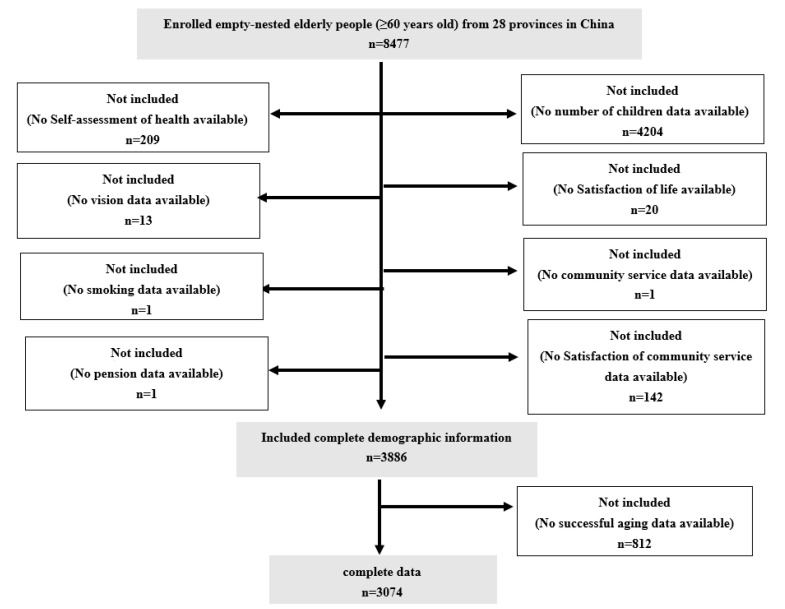
Sample selection Flowchart.

In the CHARLS data, there were 8477 empty-nesters aged 60 and above. According to the research needs, a total of 3,074 empty-nesters were included after excluding the data with missing values in demographic data and successful aging data (Figure 1). Table 1 demonstrates the demographic characteristics of the respondents and shows the influence of individual, family, and social characteristics on the successful aging of empty-nesters at three levels: microsystem, mesosystem, and macrosystem. The results showed that there were significant differences in age, gender, education, self-rated health, life satisfaction, vision, hearing, availability of financial income, sleep time, exercise, drinking, and smoking in the microsystem (*p* < 0.05), marital status, mode of residence, number of children, frequency of contact with children, financial support for children, spousal satisfaction, and child satisfaction in the mesosystem (*p* < 0.05), and place of residence, health insurance, and pension in the macrosystem (*p* < 0.05).

### 3.2. Percentage of Participants Meeting the Criteria of Successful Aging

Table 2 shows the rate of successful aging and its five elements. The results showed a successful aging rate of 5.9% in this study (*n* = 180). The percentages of no disease, high cognition functioning, no depression, active engagement with life, and no functional disability were 43.3%, 50.5%, 49.9%, 49.4%, and 68.6%, respectively.

### 3.3. The Factors Associated with Successful Aging

The multivariate logistic regression analysis in Table 3 controlling for microsystem, mesosystem, and macrosystem related factors shows the influencing factors of successful aging. Successful aging was used as the dependent variable, and variables that were significant for the univariate analysis were included in the multivariate logistic regression analysis.

In the microsystem, in comparison with those older adults aged 80 years and over, empty-nesters aged 60–69 years (odds ratio [OR] 3.157, 95% confidence interval [CI] 1.332–7.481) and 70–79 years (OR 2.870, 95% CI 1.201–6.860) were more likely to achieve successful aging. Relative to those with illiterate education, empty-nesters at middle school and above (OR 10.123, 95% CI 4.732–21.658) had significantly greater odds of aging successfully. Empty-nesters with good self-rated health (OR 6.348, 95% CI 3.569–11.294) had significantly greater odds of successful aging compared to older adults with poor self-rated health. Older adults with good life satisfaction (OR 3.157, 95% CI 1.227–8.125) were more likely to achieve successful aging compared to older adults with poor life satisfaction. Empty-nesters with financial income (OR 1.848, 95% CI 1.210–2.822) were more likely to age successfully compared to those without financial income. Empty-nesters with better hearing (OR 1.970, 95% CI 1.064–3.646) were more likely to age successfully compared to older adults with poor hearing. In the mesosystem, successful aging is more likely to be achieved in empty-nesters with children who they meet once in less than or equal to 3 months (OR 1.627, 95% CI 1.001–2.644) than with children who they meet once in more than 3 months. In the macrosystem, the elderly with urban employee medical insurance (OR 2.345, 95% CI 1.014–5.422) are more likely to achieve successful aging than those without medical insurance.

## 4. Discussion

This study is an important attempt to explore the successful aging of empty-nesters based on the social ecosystem theory [24]. The results show that the level of successful aging of Chinese empty-nesters is low and the correlation factors are age, culture, income, self-rated health, life satisfaction, and hearing at the microsystem level of the social-ecological system. We also innovatively found that the frequency of contact with children at the mesosystem level and medical insurance at the macrosystem level play an important role in the realization of successful aging.

In this study, the successful aging rate of the empty-nesters in China was 5.9%, which is lower than the successful aging rates of older adults in other studies in China (14.41% [20], 13.2% [19], and also lower than the successful aging rates of older adults in East Asian countries such as Japan (29.2%) and Korea (25.5%) [30]. This may be related to the fact that the sample selected for this study was empty-nesters. The long-term lack of family care and spiritual comfort among the empty-nesters elderly due to the absence of their children from their surroundings can easily induce various physical and mental problems [15], thus reducing their quality of life and affecting the achievement of successful aging. In addition, the subjects included in this study were from rural areas (76.2%), who had relatively lower health awareness and limited available medical resources compared with the urban elderly, so they were more likely to suffer from diseases, thus affecting the realization of successful aging [30]. Among the five key elements that make up successful aging, 43.3% of empty-nesters are free of disease, 50.5% of empty-nesters have a high cognitive function, 49.9% of empty-nesters are free of depressive symptoms, 68.6% of empty-nesters are free of functional impairment, and 49.4% of empty-nesters can actively participate in society. The element of no chronic disease had the lowest percentage of empty-nesters among the five elements, and this result was consistent with previous studies [19].

When it comes to influencing factors, in the microsystem of social-ecological systems theory [24], age has a significant impact on successful aging. Younger empty-nesters have higher levels of successful aging, which is consistent with the results of previous studies [8,19,20]. Previous studies have even shown that the likelihood of successful aging decreases to 0.64 times for every 5 years of age increase [31]. Consistent with the results of previous studies [8,19,20,32], higher levels of education and economic resources increased the likelihood of achieving successful aging for empty-nesters. Previous studies have even shown that for every 1 level increase in education, the rate of successful aging increases by 1.4 times [33]. Older adults with higher education levels have better social and economic status, medical services, and social participation than those with lower education levels, so they have more opportunities to receive external information, which makes them more aware of health perception and management, and have more sense of the meaning of life, better the self-esteem [21] and the higher the life satisfaction [34] of older adults will be, thus they are better adapted to the aging process [32]. Among several health-related factors in the microsystem, hearing, self-rated health, and life satisfaction all had an impact on successful aging among empty-nesters. First, the hearing function is associated with successful aging in empty-nesters, consistent with the findings of Liu et al. [7]. WHO data in 2018 showed that hearing disability has become the most common type of disability in the elderly, and about one-third of the elderly aged 65 years and over worldwide have disabling hearing impairment [35]. Hearing loss not only makes it difficult for older adults to receive information and language communication, but also leads to psychological problems such as loneliness and depression, which significantly inhibit their social participation and thus affect their quality of life and ability to perform daily activities [36]. Empty-nesters are more likely to experience social isolation due to hearing impairment and thus a series of physical and psychological problems. Second, empty-nesters who rated themselves in good health have lower rates of morbidity and better mental and physical health than empty-nesters who rated themselves in poor health, and therefore more likely to achieve successful aging, this result is consistent with previous studies [8]. In addition, life satisfaction was positively associated with successful aging. Life satisfaction is a measure of a person’s perceived happiness level [37], which is an important psychological factor reflecting the mental health and quality of life of the elderly [38], and has a predictive role in the successful aging of the elderly.

We exploratively found that in addition to the microsystem level in the social-ecological system theory mentioned above, the frequency of contact with children in the mesosystem is also an important influencing factor for the successful aging of empty-nesters. It has been noted [39] that for older adults, good social functioning appears to be more conducive to promoting life satisfaction than physical functioning, which fits with socioemotional selectivity theory [40], which suggests that as they age, older adults become progressively less mobile and prone to dysphoria, and as they become increasingly aware of the limited time they have left, they seek meaningful intimate relationships. In addition, the stress buffer hypothesis model [41] also mentions that social support may also have an indirect protective effect on older adults by buffering the adverse effects of depressive symptoms on life satisfaction. In China, the family is the basic unit of individual life, as pointed out in the social convey model [42], the closest social partners of the elderly mainly come from their primary family members (spouses and children), when they live with their spouses or children, they can not only provide the elderly with the emotional support they need, but also take care of them in their daily life, which plays a pivotal role in maintaining the psycho-physical health of the elderly. Having children’s support and the more frequent exchange of ideas and emotions with children, will help empty-nesters to have a sufficient sense of belonging and improve their self-confidence and self-efficacy, and enhance their ability to cope with emergencies and illnesses, thus making it easier for them to achieve physical and mental health, which is an important condition to enhance their life satisfaction and thus achieve successful aging. So in recent years, more and more studies have focused on social factors, such as the determinants of social capital on the health of older adults [43]. Therefore, children should give more care and companionship to the elderly. Single empty-nesters can be encouraged to choose another spouse. The architectural structure can also be improved to establish a “separate but not separate” architectural layout between children and the elderly, to alleviate the psychological loneliness and insecurity of the rural empty-nesters. Secondly, recommendations include focusing on the community function to establish a community support system for empty-nesters, strengthen interactions between neighbors, and form a new model of mutual assistance for the elderly.

Finally, empty-nesters with urban employee medical insurance in the macrosystem have greater odds of achieving successful aging. Other types of medical insurance are not associated with successful aging, probably because urban employees’ medical insurance has the highest reimbursement rate for the elderly, which can effectively prevent empty-nesters from delaying or avoiding medical treatment due to high medical costs, and is more conducive to promoting the health of the elderly. This again reflects the idea that literacy and economic income have an impact on the successful aging of empty-nesters. Higher literacy levels lead to higher income from formal employment and employee health insurance, resulting in better physical and psychological conditions, and a higher quality of life, thus contributing to successful aging [44,45]. In this study, the proportion of empty-nesters with chronic diseases (56.7%) is high, so the need for long-term medication will incur huge medical costs. In order to save money and reduce the financial burden on their children, empty-nesters may take the initiative to stop taking a certain medication without the supervision of their children, reduce the frequency or dose of medication, or replace it with a more economical medication. Medicare can help the elderly bear some of the treatment costs during the treatment process, which has positive significance in improving medication compliance and the prognosis of the disease. Therefore, the relevant departments must increase the publicity of medical insurance, improve the recognition of medical insurance among the elderly, increase the medical reimbursement ratio, simplify the reimbursement process, balance the allocation of urban and rural medical and health resources, and focus on vulnerable elderly groups such as empty-nesters, so that the elderly can have access to medical care and thus achieve successful aging.

## 5. Limitations

However, there are some limitations to this study. First, because this study is a cross-sectional study, the causal relationship between successful aging and each variable cannot be inferred, and more longitudinal studies are needed in the future to explore the causal relationship to formulate intervention measures more accurately. Second, this study is secondary data, the memory bias of the respondents and the loss of some respondents cause the data to have missing values, thus affecting the representativeness of the study sample. Finally, the CHARLS cohort was not originally designed to explore successful aging, so the lack of variables that may influence successful aging, such as self-efficacy and coping styles, can bias the results somewhat. Therefore, these factors could be further explored in future studies. However, this study is an important exploration of the successful aging of empty-nesters and provides a certain theoretical basis for the corresponding policy formulation.

## 6. Conclusions

In summary, the successful aging rate of empty-nesters is low. The factors associated with successful aging include several microsystem variables, namely age, education, individual income, hearing, self-rated health, and life satisfaction. The mesosystem variable is the frequency of contact with children and the macrosystem variable is the type of medical insurance. We should also pay attention to the influence of family and society on the successful aging of the empty-nesters, encourage children to give more care and companionship to empty-nesters to avoid loneliness among the elderly, hold corresponding senior activities in the community to improve the social participation of the elderly, and improve national medical security system to enhance the happiness of the empty-nesters, to achieve successful aging.

## Figures and Tables

**Table 1:** Characteristics of participants and factors related to successful aging (*n* = 3074).

Level	Variables	Categories	Total Number Survey (*n* (%))	Number of SA	Proportion of SA (%)	χ^2^
Microsystem	Age	<70	1652 (53.7)	115	7.00	13.594 **
		70–79	1097 (35.7)	59	5.40	
		≥80	325 (10.6)	6	1.80	
	Gender	Male	1471 (47.9)	103	7.00	6.726 *
		Female	1603 (52.1)	77	4.80	
	Education	Literate	924 (30.1)	8	0.90	102.623 ***
		Primary and below	1433 (46.6)	81	5.70	
		Junior high school and above	717 (23.3)	91	12.70	
	Self-rated health	Good	485 (15.8)	59	12.20	81.031 ***
		Fair	1404 (45.7)	103	7.30	
		Poor	1185 (38.5)	18	1.50	
	Satisfaction for life	Satisfaction	1022 (33.2)	65	6.40	20.743 ***
		Fair	1615 (52.5)	110	6.80	
		Dissatisfaction	437 (14.2)	5	1.10	
	Vision	Good	607 (19.8)	60	9.90	31.231 ***
		Fair	1543 (50.2)	92	6.00	
		Poor	923 (30.0)	28	3.00	
	Hearing	Good	784 (25.5)	78	9.90	37.892 ***
		Fair	1709 (55.6)	88	5.10	
		Poor	581 (18.9)	14	2.40	
	Individual income	Have	287 (9.3)	36	12.50	25.685 ***
		Have not	2787 (90.7)	144	5.20	
	Sleep	≥7 h	1153 (37.5)	86	7.50	8.624 **
		<7 h	1921 (62.5)	94	4.90	
	Exercise	Yes	2695 (87.7)	172	6.40	11.054 **
		No	380 (12.3)	8	2.10	
	Smoking	Still Have	756 (24.6)	60	7.90	8.408 *
		Quit	591 (19.2)	27	4.60	
		Never	1727 (56.2)	93	5.40	
	Drinking	Drink more than once a month	673 (21.9)	58	8.60	12.267 **
		Drink but less than once a month	218 (7.1)	13	6.00	
		Never	2183 (71.0)	109	5.00	
Mesosystem	Marital status	Married	2131 (69.3)	144	6.80	11.119 *
		Separated	12 (0.4)	0	0.00	
		Divorced	53 (1.7)	3	5.70	
		Widowed	832 (27.1)	32	3.80	
		Never married	46 (1.5)	1	2.20	
	Living Mode	Live with spouse	2132 (69.3)	144	6.80	10.231 **
		Living alone	943 (30.7)	36	3.80	
	Number of children	0	58 (1.9)	1	1.70	35.492 ***
		1	249 (8.1)	29	11.60	
		2	771 (25.1)	65	8.40	
		≥3	1997 (65.0)	85	4.30	
	Number of siblings	0	123 (4.0)	8	6.50	0.107
		1	224 (7.3)	13	5.80	
		2	426 (14.2)	25	5.70	
		≥3	2292 (74.6)	134	5.80	
	Contact with child (Month/time)	>3	584 (19.0)	21	3.60	6.678 *
		≤3	2490 (81.0)	159	6.40	
	Financial support from children	Have	2706 (88.0)	161	5.90	0.378
		Have Not	369 (12.0)	19	5.10	
	Financial support to children	Have	1266 (41.2)	94	7.40	9.641 **
		Have Not	1809 (58.8)	86	4.80	
	Satisfaction with spouse	Satisfaction	1038 (33.8)	71	6.80	11.998 **
		Fair	1045 (34.0)	72	6.90	
		Dissatisfaction	301 (9.8)	12	4.00	
		No spouse	690 (22.4)	25	3.60	
	Satisfaction with child	Satisfaction	1593 (51.8)	102	6.40	8.049 *
		Fair	1224 (39.8)	73	6.00	
		Dissatisfaction	191 (6.2)	4	2.10	
		No child	66 (2.1)	1	1.50	
Macrosystem	Community type	Non-rural	731 (23.8)	75	10.30	33.748 ***
		Rural	2343 (76.2)	105	4.50	
	Ethnicity	Han	2881 (93.7)	171	5.90	0.554
		Minority	193 (6.3)	9	4.60	
	Religious belief	Have	363 (11.8)	20	5.50	0.097
		Have Not	2711 (88.2)	160	5.90	
	Medical insurance	Urban employee medical insurance	438 (14.2)	57	13.00	49.75 ***
		Urban and rural resident medical insurance	498 (16.2)	30	6.00	
		New rural cooperative medical insurance	1948 (63.4)	86	4.40	
		None	191 (6.2)	7	3.70	
	Community care service	Have	662 (21.5)	34	5.10	0.793
		Have Not	2412 (78.5)	146	6.10	
	Community medical satisfaction	Satisfaction	1271 (41.3)	62	4.90	4.367
		Usual	1228 (39.9)	84	6.80	
		Dissatisfaction	575 (18.7)	34	541.00	
	Medical aid	Have	17 (0.6)	0	0.00	1.063
		Have not	3057 (99.4)	180	5.90	
	Pension	Pension for Public Servants	625 (20.3)	68	10.90	37.961 ***
		Urban and Rural Resident Pension	2028 (66.0)	99	4.90	
		None	421 (13.7)	13	3.10	

* *p* < 0.05, ** *p* < 0.01, *** *p* < 0.001.

**Table 2:** Percentage of participants meeting the criteria of successful aging.

Variables	*n* (%)
Successful aging	180 (5.9)
No disease	1330 (43.3)
High cognitive functioning	1553 (50.5)
No depression	1533 (49.9)
No disability	2108 (68.6)
Active engagement with life	1520 (49.4)

**Table 3:** Multiple logistic regression for successful aging.

Level	Variables	Categories	B	SE	Wald χ2	*p*	OR (95%CI)
Microsystem	Age (≥80)				6.825	0.033	
		<70	1.150	0.44	6.82	<0.01	3.157 (1.332–7.481)
		70–79	1.054	0.445	5.624	<0.05	2.870 (1.201–6.860)
	Education (Literate)				38.486	<0.001	
		Primary and below	1.708	0.377	20.521	<0.001	5.519 (2.636–11.557)
		Junior high school and above	2.315	0.388	35.59	<0.001	10.123 (4.732–21.658)
	Individual income (Have not)	Have	0.614	0.216	8.067	<0.01	1.848 (1.210–2.822)
	Hearing (Poor)	Good	0.678	0.314	4.652	<0.05	1.970 (1.064–3.646)
		Fair	0.303	0.304	0.997	0.318	1.354 (0.747–2.456)
	Self-rated health (Poor)				39.597	<0.001	
		Good	1.848	0.294	39.56	<0.001	6.349 (3.569–11.249)
		Fair	1.316	0.266	24.52	<0.001	3.729 (2.215–6.278)
	Satisfaction of life (Dissatisfaction)				7.551	<0.05	
		Satisfaction	1.15	0.482	5.684	<0.05	3.157 (1.227–8.125)
		Fair	1.282	0.472	7.38	<0.01	3.606 (1.429–9.095)
Mesosystem	Contact with child (>3)	≤3	0.487	0.248	3.859	<0.05	1.627 (1.001–2.644)
Macrosystem	Medical insurance (None)				8.072	<0.05	
		Urban employee medical insurance	0.852	0.428	3.973	<0.05	2.345 (1.014–5.422)
		Urban and rural resident medical insurance	0.455	0.45	1.024	0.312	1.577 (0.653–3.810)
		New rural cooperative medical insurance	0.339	0.422	0.645	0.422	1.403 (0.614–3.207)

Note: CI = confidence interval; OR = odds ratio; SE = standard error.

## Data Availability

The technical appendix, dataset creation plan/protocol and statistical code are available from the first author.

## Supplementary Materials

The following supporting information can be downloaded at: https://www.mdpi.com/article/10.3390/ijerph191911885/s1. Table S1: RECORD Checklist.
